# A. V. M. A.

**Published:** 1901-10

**Authors:** 


### A. V. M. A.

THE following attended the Thirty-eighth Annual Meeting of the American Veterinary Medical Association at Atlantic City, N. J., September 2 to 6, inclusive, 1901.

MEMBERS.

Francis Abele, Jr., 8 Spear Street, Quincy, Mass.; E. B. Ackerman, 167 Clymer Street, Brooklyn, N. Y.; F. E. Anderson, Findlay, Ohio; W. J. Armour, Goshen, Indiana; Edward C. Beckett, 547 Albany Street, Boston, Mass.; Roscoe R. Bell, Seventh Avenue and Union Street, Brooklyn, N. Y.; George H. Berns, 74 Adams Street, Brooklyn, N. Y.; Thomas Bland, Waterbury, Conn.; T. A. Bray, El Paso, Texas; S. Brenton, 85 Fifth Street, Detroit, Mich.; T. Earle Budd, 90 Park Street, Orange, N. J.; Alexander Burr, Old Court House, Boston, Mass.; Tait Butler, Raleigh, N. C.; C. E. Clayton, New York City; A. S. Cooley, 1184 E. Madison Avenue, Cleveland, Ohio; T. Bent Cotton, Mount Vernon, Ohio; Albert, E. Cunningham, 215 Huntington Street, Cleveland, Ohio; Cooper Curtice, Kingston, R. I.; W. H. Dalrymple, Baton Rouge, La.; J. F. Devine, Rhinebeck, N. Y.; W. H. Dodge, Leominster, Mass.; William Dougherty, 1035 Cathedral Street, Baltimore, Md.; Robert W. Ellis, 453 W. 150th Street, New York, N. Y.; A. A. Etienne, St. Hyacinthe, Province of Quebec, Canada; H. P. Eves, Wilmington, Del.; A. M. Farrington, 1436 Chapin Street, Washington, D. C.; Langdon Frothingham, 20 Hereford Street, Boston, Mass.; Carl W. Gay, Syracuse, N. Y.; J. T. Glennon, 119 Plane Street, Newark, N. J.; Charles T. Goentner, Bryn Mawr, Pa.; L. Kenneth Green, 550 Second Avenue, Detroit, Mich.; John W. Groves, 40 York Street, Hamilton, Ontario; R. W. Hewitt, West Philadelphia Stock Yards Hotel, Philadelphia, Pa.; H. D. Hanson, 160 Eldridge Street, New York City, N. Y.; S. J. J. Harger, 205 N. Twentieth Street, Philadelphia, Pa.; W. F. Harrison, Bloomfield, N. J.; Samuel G. Hendren, 30 Plane Street, Newark, N. J.; W. F. Heyde, 1215 S. Jefferson Avenue, St. Louis, Mo.; F. F. Hoffman, Brookville, Pa.; Herbert Hoopes, Bynum, Md.; J. B. Hopper, Ridgewood, N. J.; W. Horace Hoskins, 3452 Ludlow Street, Philadelphia, Pa.; Joseph Hughes, 2537 State Street, Chicago, Ill.; Rush S. Huidekoper, 1304 Sansom Street, Philadelphia, Pa.; M. Jacobs, Knoxville, Tenn.; G. A. Johnson, Exchange Building, Stock Yards, Sioux City, Iowa; William Henry Kelly, 233 Western Avenue, Albany, N. Y.; G. C. Kesler, Holley, N. Y.; W. B. Kille, Salem, N. J.; M. E. Knowles, Helena, Montana; J. V. Laddey, Arlington, N. J.; James Law, Cornell University, Ithaca, N. Y.; E. L. Loblein, New Brunswick, N. J.; William Herbert Lowe, Paterson, N. J.; J. Payne Lowe, Passaic, N. J.; Charles C. Lyford, 817 Third Avenue, South Minneapolis, Minn.; Richard P. Lyman, Hartford, Conn.; F. W. McLellan, Bridgeport, Conn.; J. C. McNeill, Department of Public Safety, Pittsburg, Pa.; C. J. Marshall, 2004 Pine Street, Philadelphia, Pa.; Wm. H. Martenet, 1005 West North Avenue, Baltimore, Md.; L. A. Merillat, 2127 Indiana Avenue, Chicago, Ill.; J. C. Meyer, 1111 Walnut Street, Cincinnati, Ohio; John R. Mohler, U. S. Inspector, Washington, D. C.; R. C. Moore, 1404 Holmes Street,

Kansas City, Mo.; G. E. Nesom, Clemson College, S. C.; James B. Paige, Amherst, Mass.; Austin Peters, 35 Congress Street, Boston, Mass.; Benj. D. Pierce, Springfield, Mass.; E. C. Porter, New Castle, Pa.; Lemuel Pope, Jr., Portsmouth, N. H.; M. M. Poucher, Oswego, N. Y.; E. M. Ranck, 422 N. Forty-first Street, Philadelphia, Pa.; James B. Rayner, 135 E. Gay Street, West Chester, Pa.; John J. Repp, Ames, Iowa; M. H. Reynolds, St. Anthony Park, Minn.; W. L. Rhoads, Lansdowne, Pa.; W. H. Ridge, Trevose, Pa.; J. C. Robert, Agricultural College, Miss.; James L. Robertson, 409 Ninth Avenue, New York City, N. Y.; Howard P. Rogers, 9 Ashford Street, Allston, Boston, Mass.; Frederick P. Ruhl, Fairmont, W. Va.; J. E. Ryder, 1634 Broadway, New York City, N. Y.; D. E. Salmon, Chief of Bureau of Animal Industry, Washington, D. C.; Thomas E. Smith, 309 Barrow Street, Jersey City, N. J.; E. H. Shepard, 793 Doan Street, Cleveland, Ohio; T. G. Sherwood, 107 W. Thirty-seventh Street, New York City, N. Y.; S. Stewart, Kansas City, Mo.; Wm. J. Tomlinson, Williamsport, Pa.; J. P. Turner, 910 O Street, Washington, D. C.; L. Van Es, Mobile, Ala.; A. G. Vogt, 119 Plane Street, Newark, N. J.; George R. White, 316 N. Front Street, Nashville, Tenn.; J. F. Winchester, Lawrence, Mass.; C. R. Witte, New Britain, Conn.

### NEW MEMBERS.

C. B. Ainsworth, Greensburg, Ind.; G. Alaire, L'Epiphaine, Province of Quebec; U. S. G. Bieber, Kutztown, Pa.; S. D. Brimhall, 2304 Emerson Street, Minneapolis, Minn.; J. M. Carter, 215 S. Seventeenth Street, Philadelphia, Pa.; William P. Fink, 127 N. Indiana Avenue, Atlantic City, N. J.; Wm. Gall, Matawan, N. J.; Joseph P. Grogan, 909 Ashland Avenue, Baltimore, Md.; J. G. Hill, Jacksonville, Fla.; T. B. Hillock, Columbus, Ohio; C. H. Jewell, Dunkirk, N. Y.; Bassett Kirby, Woodbury, N. J.; L. M. Land, Lexington, Ky.; Harry Lukes, Springfield, Mass.; William Lukes, Springfield, Mass.; R. W. McCulley, 160 E. Twenty-fourth Street, New York City, N. Y.; Elijah Matthews, Jersey City, N. J.; V. A. Moore, Ithaca, N. Y.; E. H. Newcomer, Mount Joy, Pa.; Charles H. Perry, 82 Park Avenue, Worcester, Mass.; D. A. Piatt, 19 W. Short Street, Lexington, Ky.; T. E. Robinson, Westerly, R. I.; Charles R. Simpson, Somerville, Mass.; D. King Smith, Ontario Veterinary College, Toronto, Canada; Stuart Thayer, Dunham, Quebec; L. E. Tuttle, Bernardsville, N. J.; B. M. Underhill, Media, Pa.

### VISITING VETERINARIANS.

F. L. Adams, Jamaica Plain, Mass.; A. Barradell, Pawling, N. Y.; James Beatty, Bureau of Animal Industry, Philadelphia, Pa.; H. G. Black, Atlantic City, N. J.; Charles W. Boyd, 26 S. Diamond Street, Allegheny, Pa.; A. S. V. Brenton, 85 Fifth Street, Detroit, Mich.; M. E. Conard, 1823 Filbert Street, Philadelphia, Pa.; J. F. Connor, Uniontown, Ala.; George T. Crowley, New Britain, Conn.; J. M. Everitt, Hackettstown, N. J.; George S. Fuller, Philadelphia, Pa.; W. W. Gardiner, Moorestown, N. J.; J. O. George, 6 S. Fifth Street, Camden, N. J.; G. F. Harker, Trenton, N. J.; S. E. Hershey, Keyser, W. Va.; A. J. Hillock, 204 E. Long Street, Columbus, Ohio; W. C. Holden, Delphos, Ohio; L. P. Hurley, Hopewell, N. J.; H. S. Jackson, Sewickley, Pa.; J. B. Jones, 1811

Atlantic Avenue, Atlantic City, N. J.; Zeno S. Keil, Perkasie, Pa.; D. R. Kohler, Boyertown, Pa.; J. R. Kuhns, Dover, Del.; J. E. Layton, Bridgeville, N. J.; J. H. Ludy, St. Albans, Vt.; A. N. Lushington, 1101 Fifth Avenue, Lynchburg, Va.; A. A. McDonnell, N. Adams, Mass.; George F. McGuire, New Britain, Conn.; Charles E. Magill, Haddonfield, N. J.; Robert S. MacKellar, Nyack, N. Y.; W. H. Mattson, Camp Ground, Pa.; James M. Mecray, Maple Shade, N. J.; J. C. Michener, Colmar, Pa.; T. F. Mullaney, 1920 Locust Street, Kansas City, Mo.; Otto G. Noack, 54 S. Sixth Street, Reading, Pa.; C. H. Olcott, New Britain, Conn.; J. F. Olweiler, Elizabethtown, Pa.; J. H. Oyler, Harrisburg, Pa.; H. F. Palmer, 235 Alexandrine Avenue, Detroit, Mich.; N. Rectenwald, 89 Washington Avenue, Pittsburg, Pa.; D. Roberts, Waukesha, Wis.; F. H. Schneider, 3512 N. Eleventh Street, Philadelphia, Pa.; A. J. Sellers, Camden, N. J.; Albert Fricke Schrieber, Sixty-second and Elmwood Avenue, Philadelphia, Pa.; F. N. Sherrick, 211 E. Apple Street, Connellsville, Pa.; James A. Sheedy, St. Albans, Vt.; W. M. Simpson, Malden, Mass.; William Simpson, Springfield, Mass.; George A. Smith, Frostburg, Md.; W. R. Smith, West Brookfield, Mass.; Frederick Stehle, 4272 Ridge Avenue, Philadelphia, Pa.; Thomas Thacker, Renfrew, Ontario, Canada; R. A. Toomey, Springfield, Mass.; S. C. Tremaine, Bridgeton, N. J.; Jesse A. Viles, 14 Coral Street, Lowell, Mass.; F. Whitney, Oswego, N. Y.; A. M. Wray, Richmond, Ill.

### OTHER VISITORS.

Charles Auerbach, 262 N. Thirteenth Street, Philadelphia, Pa., representing West Disinfecting Company; R. J. Burton, care H. K. Mulford Company, Philadelphia, Pa.; J. A. Cadmus, Passaic, N. J.; Hon. Franklin Dye, Secretary State Tuberculosis Commission, Trenton, N. J.; Zan Cotter, Chicago, Ill.; T. E. Crossman, Official Stenographer, 1829 Park Row Building, New York City, N. Y.; A. R. Davison, representing E. R. Squibb & Sons, New York City, N. Y.; Alexander Eger, veterinary and medical book publisher, 34 Van Buren Street, Chicago, Ill.; Master T. B. Hillock, Ohio; L. D. Horner, Woodstown, N. J.; Master Cheston Hoskins, Philadelphia, Pa.; J. R. Johns, M. D., representing Hance Brothers & White, Philadelphia, Pa.; S. E. Little, Bernardsville, N. J.; the Honorable Henry C. Loudenslager, of New Jersey (Paulsboro); A. J. McCloskey, Chestnut Hill, Philadelphia, Pa.; Cynes Mantz, of the Kretol Chemical Company, Washington, D. C.; Hon. Franklin P. Stoy, Mayor of Atlantic City, Atlantic City, N. J.; the Honorable Joshua S. Salmon, of New Jersey (Boonton); George W. Teufel, 114 S. Tenth Street, Philadelphia, Pa.; James Truman, 4505 Chester Avenue, Philadelphia, Pa.

### LADIES.

Mrs. E. B. Ackerman, Brooklyn, N.Y.; Mrs. C. B. Ainsworth, Greensburg, Ind.; Mrs. F. E. Anderson, Findlay, Ohio; Mrs. M. D. Bell, Philadelphia, Pa.; Mrs. R. R. Bell and two children, Brooklyn, N. Y.; Mrs. G. H. Berns and children, New York, N. Y.; Miss Nellie Berns, New York, N.Y.; Mrs. Thomas Bland, Waterbury, Conn.; Miss Ethel Bland, Waterbury, Conn.; Mrs. Tait Butler, Raleigh, N. C.; Mrs. T. A. Bray, El Paso, Texas; Mrs. S. Brenton and Miss Brenton, Detroit, Mich.; Mrs. T. E. Budd, Orange, N. J.;

Miss Helen R. Budd, Orange, N. J.; Mrs. Flora H. Cooley, Cleveland, Ohio; Miss C. E. Clark, New York, N. Y.; Mrs. T. E. Crossman, New York, N. Y.; Mrs. Alex. Eger, Chicago, Ill.; Mrs. L. K. Green, Detroit, Mich.; Mrs. J. O. George, Camden, N. J.; Miss George, Camden, N. J.; Mrs. Charles T. Goentner, Bryn Mawr, Pa.; Mrs. John Groves, Hamilton, Ont.; Mrs. Joseph Hughes, Chicago, Ill.; Mrs. H. D. Hanson, New York, N. Y.; Mrs. Mary J. Hart, Atlantic City, N. J.; Mrs. W. Horace Hoskins, Philadelphia, Pa.; Mrs. W. G. Huyett, Minersville, Pa.; Mrs. J. B. Jones, Atlantic City, N. J.; Mrs. G. A. Johnson, Sioux City, Iowa; Mrs. T. B. Hillock, Columbus, Ohio; Mrs. W. H. Kelly, Albany, N. Y.; Mrs. J. Payne Lowe, Passaic, N. J.; Mrs. J. H. Ludy, Watkins, Vt.; Mrs. Harry Lukes, Springfield, Mass.; Mrs. E. S. Loblein, New Brunswick, N. J.; Mrs. C. J. Marshall, Philadelphia, Pa.; Mrs. J. R. Mohler, Washington, D. C.; Mrs. W. H. Lowe, Paterson, N. J.; Mrs. L. A. Merillat, Chicago, Ill.; Mrs. E. H. Newcomer, Mt. Joy, Pa.; Mrs. G. E. Nesom, Clemson College, S. C.; Mrs. D. A. Piatt, Lexington, Ky.; Mrs. J. B. Rayner, West Chester, Pa.; Mrs. W. H. Ridge, Trevose, Pa.; Mrs. D. Roberts, Waukesha, Wis.; Mrs. F. P. Ruhl, Fairmont, W. Va.; Mrs. J. E. Ryder, New York, N. Y.; Mrs. E. M. Ranck, Philadelphia, Pa.; Mrs. John J. Repp, Ames, Iowa; Mrs. W. Runge, Newark, N. J.; Mrs. James A. Sheedy, St. Albans, Vt.; Mrs. C. P. Simpson, Somerville, Mass.; Mrs. S. Stewart, Kansas City, Mo.; Mrs. R. A. Toomey, Springfield, Mass.; Mrs. J. P. Turner, Washington, D. C.; Mrs. George R. White, Nashville, Tenn.; Miss Wilson, Chesterville, Pa.

### OFFICERS

*For the Ensuing Year*, 1901–1902.

*President*—J. F. Winchester, Lawrence, Mass.
*First Vice-President*—R. R. Bell, Brooklyn, N. Y.
*Second Vice-President*—W. H. Dalrymple, Baton Rouge, La.
*Third Vice-President*—M. E. Knowles, Helena, Mont.
*Fourth Vice-President*—M. H. Reynolds, St. Anthony Park, Minn.
*Fifth Vice-President*—Wm. Dougherty, Baltimore, Md.
*Secretary*—S. Stewart, Kansas City, Mo.
*Treasurer*—Wm. Herbert Lowe, Paterson, N. J.

### COMMITTEES.

*Executive.*—D. E. Salmon, District of Columbia, *Chairman;* H. D. Hanson, New York; M. H. Reynolds, Minnesota; C. A. Carey, Alabama; G. A. Johnson, Iowa; W. H. Hoskins, Pennsylvania; T. E. Budd, New Jersey.

*Disease.*—E. M. Ranck, Pennsylvania, *Chairman;* James Law, New York; A. T. Peters, Nebraska; L. Frothingham, Massachusetts; R. R. Dinwiddie, Arkansas.

*Intelligence and Education.*—E. B. Ackerman, New York, *Chairman;* T. B. Cotton, Ohio; W. J. Hinman, Massachusetts; G. W. Dumphy, Michigan; J. C. Roberts, Mississippi.

*Finance.*—R. C. Moore, Missouri, *Chairman;* W. H. Kelly, New York; C. Curtis, Rhode Island.

*Publication.*—M. H. Reynolds, Minnesota, *Chairman;* R. R. Bell, New York; J. J. Repp, Iowa; L. Van Es, Alabama; R. P. Lyman, Connecticut.

*Resolutions.*—Tait Butler, North Carolina, *Chairman;* D. E. Salmon, District of Columbia; S. Brenton, Michigan; S. B. Nelson, Washington; G. H. Berns, New York.

*Army Legislation.*—L. Pearson, Pennsylvania, *Chairman;* R. S. Huidekoper, Pennsylvania; W. H. Lowe, New Jersey; A. Peters, Massachusetts; M. E. Knowles, Montana.

### RESOLUTIONS

passed at the Thirty-eighth Annual Meeting of the American Veterinary Medical Association, Hotel Rudolf, Atlantic City, September 4, 1901.

### Bovine Tuberculosis.

*Resolved,* That the interest now manifested by the general public in the subject of bovine tuberculosis is fully justified by the prevalence of the disease and the tendency to rapid increase among cattle and extension to swine which it has shown during recent years.

*Resolved,* That the evidence of accidental inoculations and clinical observation apparently demonstrated that bovine tuberculosis may be communicated to man, and that in the opinion of this Association the British Congress did well in finding that "medical officers of health should continue to use all the power at their disposal and relax no efforts to prevent the spread of tuberculosis in milk and meat."

*Resolved,* That experiments showing the difficulty or the impossibility of transmitting human tuberculosis to cattle in a fatal form cannot be accepted as evidence that the bovine bacillus, which is far more virulent and fatal for many animals, cannot infect man. On the contrary, the greater disease-producing power of the bovine bacillus would appear to make it more dangerous for human beings as well as for lower animals.

*Resolved,* That the evidence showing the practical impossibility of cattle being infected with human tuberculosis under ordinary conditions is a great encouragement for the eradication of bovine tuberculosis, since it proves that the danger so often feared that cattle if freed from the disease would be immediately reinfected from mankind, does not exist in fact and need not be considered.

*Resolved,* That if it is admitted that human tuberculosis is not communicable to cattle under ordinary conditions, this should be a great encouragement for the eradication of bovine tuberculosis, since it would prove that the danger so often feared that cattle if freed from the disease would be immediately reinfected from mankind, does not exist in fact and need not be considered.

*Resolved,* That even if it were true that bovine tuberculosis is not communicable to man, it would nevertheless be to the interest of cattle and swine growers to have the disease eradicated as an economic measure; and such eradication would also be to the interest of the public, since it would have great effect as a means of preserving the food-supply of the nation and of protecting consumers from the unwholesome products of diseased animals.

### Army Veterinary Department.

Whereas, The bill providing for the establishment of a suitable Army Veterinary Department, giving its members the rank of commissioned officers, was defeated in the Senate, after having once been passed in the Senate and House; therefore, be it

*Resolved*, That this Association deplores the defeat of a measure that would have placed the military veterinarian in this country on the same basis as his professional colleague in the armies of the old world, and guaranteed efficient veterinary care for animals in the United States army, representing the investment of millions of dollars of the people's money; and be it further

*Resolved*, That the thanks of this Association be extended to our friends in the Congress of the United States who supported the bill; and be it further

*Resolved*, That this Association continue its endeavors and work until there is an efficient and proper veterinary service in the United States army.

*Resolved*, That a copy of these resolutions be entered upon the records of this Association.

### Claude D. Morris.

Whereas, Dr. Claude D. Morris, a member of this Association, wrote a letter to the Secretary of War against the passage of the Army Veterinary bill, which was widely quoted, at the time it was written, in the press of the country; and

Whereas, It misrepresented the views of nearly all the members of our profession and may have produced harmful results, which may even have an effect in the future if this Association does not take a strong stand against such behavior; therefore, be it

*Resolved*, That this Committee recommend the immediate expulsion of Dr. Claude D. Morris from this Association; and be it further

*Resolved*, That the By-laws of this Association are hereby suspended in order that the said Dr. Claude D. Morris can be immediately expelled, and that he is hereby expelled from membership in this Association; and be it further

*Resolved*, That a copy of these resolutions be hereby entered on the records of this Association.

### College Catalogue Illustrations.

Whereas, This Association notes with regret the fact that certain recognized veterinary colleges of America use illustrations in their catalogues and other advertising literature that are undignified and offensive to good taste; therefore, be it

*Resolved*, That it is the sense of this Association that the college has no greater license in the matter of display advertising than is legitimately enjoyed by the individual; and be it further

*Resolved*, That the college which uses illustrations in its advertising literature for any other purpose than to show the character of its buildings and equipment is acting in violation of the spirit of our code of ethics.

### Veterinarians to Boards of Health.

Whereas, The scientific training of graduated veterinarians especially qualifies them for the duties of sanitarians and veterinarians to State and municipal boards of health; and

WHEREAS, We note with regret the fact that at present proper recognition is not given to the importance of veterinary training in the personnel of State boards of health, sanitary commissions, and other united bodies; therefore, be it

*Resolved*, That the President of this Association is hereby instructed to appoint a Committee of five, whose duty it shall be to wage a campaign of education and endeavor to secure proper recognition for the profession in the matter referred to above; and be it further

*Resolved*, That the sum of $100 is hereby appropriated out of the funds of the Association for the use of this Committee.

### AMERICAN VETERINARY PHARMACOPŒIA.

WHEREAS, There is a necessity for an American Veterinary Pharmacopœia; therefore, be it

*Resolved*, That a Committee be appointed, consisting of seven members, for the purpose of compiling a Pharmacopœia of Veterinary Medicine; be it further

*Resolved*, That on the completion of said Pharmacopœia it shall be submitted for examination and discussion, and, if found satisfactory, proper arrangements shall be taken by the Association for its publication.

### LOCAL COMMITTEE OF ARRANGEMENTS.

WHEREAS, The excellent work done by the Local Committee of Arrangements, the cordial reception by the Chief Magistrate of the city, the courtesies of the Atlantic City press, and the intense interest of the members of the Veterinary Medical Association of New Jersey have all contributed much to the pleasure and profit of our meeting; therefore, be it

*Resolved*, That this Association tender their sincere thanks and express their appreciation of the courtesies extended us, and trust that our meeting in this city may contribute much to the welfare and advancement of the profession.

---

### OBITUARY.

### J. H. STICKNEY.

WHEREAS, It has pleased Almighty God to remove from our midst, during the past year, Dr. J. H. Stickney, of Boston, Mass., the first President of the United States Veterinary Medical Association, in 1863–64. He saw it grow from the time of its inception to its present size and strength, and even took an active interest in its advancement and the welfare of its members from the time of his first connection with it until the day of his death, at the ripe age of seventy-five years. It is due to the efforts of him and his associates in the early days of the organization that it has reached its present influence and membership. May we endeavor to do as much for the generation to come; therefore, be it

*Resolved*, That this Association sincerely mourns his loss, and extends to his family its sympathy in their bereavement; and be it further

*Resolved*, That these resolutions be entered in the records of this Association and a copy be sent to his family.

### Charles Burden.

Whereas, It has pleased Almighty God to remove from our midst, during the past year, Dr. Charles Burden, of New York City, Secretary of the United States Veterinary Medical Association, 1865–67, and later its Treasurer—one of the early members of this organization to whom the profession owes so much. We regret to hear of his decease; therefore, be it

*Resolved*, That this Association sincerely mourns his loss, and extends to his family its sympathy in their affliction; and be it further

*Resolved*, That these resolutions be entered on the records of this Association and a copy be sent to his bereaved family.

### A. W. Clement.

Whereas, It has pleased Almighty God to remove from our midst, during the past year, Dr. A. W. Clement, of Baltimore, Md., President of this Association in 1898–99—a man in the prime of life, who apparently had many years of usefulness before him, but who was cut off at the age of forty-four years. We deplore his loss to his country, his State, and to this Association; therefore, be it

*Resolved*, That this Association sincerely regrets his decease so early in his useful career, and extends to his widow its sympathy in her affliction and be it further

*Resolved*, That these resolutions be entered on the records of this Association and a copy be sent to his widow.

### P. D. Coffee.

Whereas, It has pleased Almighty God to remove, during the past year, Dr. P. D. Coffee, of Svensen, Oregon, a member of this Association; therefore, be it

*Resolved*, That this Association regrets his loss and extends its sympathy to his bereaved family; and be it further

*Resolved*, That these resolutions be entered upon the records of this Association and a copy be sent to his family.

### George Fleming.

Whereas, It has pleased Almighty God to remove, during the past year, Dr. George Fleming, of England, an honorary member of this Association —a man of marked literary and scientific attainments; he was a living example of what energy, intelligence, and perseverance will do for any ambitious young man, rising from forge boy to the position of Chief Veterinarian in the British army; therefore, be it

*Resolved*, That this Association regrets his decease, and extends to his family its sympathy in their bereavement; and be it further

*Resolved*, That these resolutions be entered on the records of this Association and a copy be sent to his family.

WILLIAM WILLIAMS.

WHEREAS, It has pleased Almighty God to remove, during the past year, Prof. William Williams, of Edinburgh, Scotland, an honorary member of this Association, Principal of the New Veterinary College at Edinburgh and author of text-books, who was honored by the profession as an educator and writer; therefore, be it

*Resolved*, That this Association regrets his loss, and extends to his family its sympathy in their bereavement; and be it further

*Resolved*, That these resolutions be entered on the records of this Association and a copy be sent to his family.

J. L. THAYER.

WHEREAS, It has pleased Almighty God to remove, during the past year, J. L. Thayer, M.D., of Newton, Mass., an honorary member of this Association—son of Dr. E. F. Thayer, President of the United States Veterinary Medical Association, 1869–71—we lament his death; therefore, be it

*Resolved*, That this Association regrets his loss, and extends its sympathy to his family in their bereavement; and be it further

*Resolved*, That these resolutions be entered upon the records of this Association and a copy be sent to his family.

STATE OF MONTANA.

WHEREAS, The State of Montana has, during the past year, enacted an efficient meat- and milk-inspection law; and

WHEREAS, The success of this measure was to a great extent due to the influence of the press of Montana, especially Dr. W. G. Eggelson, editor of the Helena *Independent*, and to the personal efforts of Senator George Stanton and Representatives W. A. Hedges, J. F. Patterson, and Joseph Dixon; therefore, be it

*Resolved*, That this Association expresses its appreciation to the above-named gentlemen and to the members of the Montana Legislature who supported the bill, and of their efforts in behalf of improved sanitary laws; and be it further

*Resolved*, That these resolutions be entered upon the records of this Association, and that a copy be sent to each of the gentlemen named above.

M. H. REYNOLDS.

WHEREAS, Dr. M. H. Reynolds has taken much pains and trouble to bring a very interesting display of photographs of pathological specimens, an example that others might emulate another year; therefore, be it

*Resolved*, That this Association thanks him for the interesting exhibition he has made.

A. YOUNGBERG.

WHEREAS, Dr. A. Youngberg, of Lake Park, Minn., a member of this Association, is confined to his home by a serious illness; therefore, be it

*Resolved*, That this Association extends to him the sincere sympathy of its members for him in his affliction and their hopes for his speedy recovery; and be it further

*Resolved*, That these resolutions be entered upon the records of this Association and that a copy be sent to Dr. Youngberg.

Journal of Comparative Medicine and Veterinary Archives.

*Resolved*, That the members of this Association express their heartfelt thanks for the kindness shown to them by the Editors of the Journal of Comparative Medicine and Veterinary Archives in furnishing free for their use the services of two stenographers and typewriters.

*Committee on Resolutions:*
Austin Peters, *Chairman*,
W. H. Dalrymple,
George White,
D. E. Salmon.

### COMMITTEE REPORTS.

Presented at the Thirty-eighth Annual Meeting of the American Veterinary Medical Association, Hotel Rudolf, Atlantic City, September 3, 1901.

### Secretary's Report.

During the entire Association year culminating in this meeting the Secretary's office has been one of activity and continuous application to the interests of the Association. Earnest effort has been made to carry out all the instructions given by the Association at its meeting held in Detroit, and to promptly conduct the correspondence, giving each member of the several committees appointed official notice of his appointment, and perfecting the membership of the large number of applicants elected at the last meeting. An unusually large number of letters have been sent and received during the year, and throughout most cordial relations have been maintained.

Under direction of President Dr. Tait Butler, letters credential were issued to Prof. A. Liautard and Dr. W. H. Wray, as delegates to the British Congress on Tuberculosis, held in London, July last. Prof. A. Liautard, of Paris, France, and Prof. Dr. Theodore Kitt, of Bavaria, were notified of their election to honorary membership in this Association, and letters of acknowledgment of the courtesy duly received and placed on file.

Resident Secretaries have been unusually active during the year—have taken more interest than formerly, which indicates a more wide-spread and general interest in the affairs of our Association; and from the large number of applications for membership on file, coming from various parts of the United States and Canada, it is believed that the influence of the A. V. M. A. is recognized as being good and helpful in all parts of the continent. The fact that printed reports of the proceedings of each meeting are promptly placed in the hands of the membership makes each member feel that he has received something of value directly from the Association.

Several letters have been received from veterinarians relative to the possibility of a change of date of meeting. Several States hold State fairs in the first week in September, and the obligations of State veterinarians and also local veterinarians are such that they cannot leave the State at this time, and the question is presented as to whether or not this Association might not profitably advance the date of meeting to some time in the month of August. I would specifically mention that the States of Iowa and Nebraska hold their annual fairs on the same date fixed for our meetings. The State veterinarians and Experiment Station men find it impracticable to leave their State on this date. True it is that no date could be fixed upon which would not inconvenience some, but it is doubtless worth the Association's while to consider the feasibility of a change of date.

Notwithstanding the large expenditures incurred during the past year, the funds of the Association will permit of the prompt payment of all bills, including the reporting, publication, and distribution of the reports of this meeting.

I cannot close this report without expressing my gratitude to the Resident Secretaries and all other officials of the Association for their cordial assistance throughout the year. It is by this co-operation that the present grand meeting has been made possible.

Very respectfully submitted,

S. STEWART,
Secretary.

### REPORT OF COMMITTEE ON FINANCE.

We, your Committee on Finance, have carefully examined the accounts of the Secretary and Treasurer for the year ending September 2, 1901, and have found them to be correct and in accord with the reports of these officers. We have examined and approved bills paid during the year to the amount of $1666.46. We have also approved bills filed with the Secretary in the sum of $414.02. Total, $2080.48.

R. C. MOORE,
G. R. WHITE,
WM. E. DOUGHERTY,
*Committee.*

### REPORT OF RESIDENT STATE SECRETARY OF ALABAMA.

BY L. VAN ES, MOBILE, ALA.

As Resident Secretary for Alabama I beg to submit the following report: As my opportunity for observation is of a limited character, the facts related mostly pertain to the extreme southern part of the State. There is an ever-increasing interest in the live-stock business throughout the country, and I consider the time near at hand when veterinarians will be able to make a living in our smaller communities. As it is at present, practitioners can only succeed in the large cities and towns. This necessarily puts a limit to the number of veterinarians in the State, and constitutes a reason why attempts to organize a State Association have thus far been a failure. Another reason for this failure is the lack of interest of those already here.

As to the prevalence of various diseases in the State, I beg to say that the following are frequently met with: Tetanus, osteoporosis, glanders, Texas fever, contagious abortion, tuberculosis, infectious pleuropneumonia of the horse, strangles, and canine distemper. I may also mention that within the last two years I have witnessed sporadic outbreaks of anthrax, swine-plague, and rabies; of the latter disease two persons have died in this city during the last year. The occurrence of osteoporosis has greatly increased during the past year, but there is nothing in evidence which can explain this greater number of cases. The greatest amount of loss in live-stock is sustained by shippers of horses, through infectious pleuropneumonia. The great horse markets of the North and West seem to constitute the principal foci, where non-immune material shipped South contract the infection. This disease is a great drawback to the Southern horse trade, and sometimes assumes a very malignant type. The loss per year being considerable, it may be well to direct the attention of our General Government to the matter, in order that some means may be found to check its ravages.

Some interest has been awakened in the principal cities of the State in municipal milk- and meat-inspection, but only one city has a more or less thorough system established, mainly through the efforts of the veterinarians of our A. and M. College at Auburn.

### Report of Resident State Secretary of New Jersey.

BY J. PAYNE LOWE, PASSAIC, N. J.

The veterinary profession of New Jersey is very glad and proud to have the opportunity to welcome the American Veterinary Medical Association within the confines of our State. Never before have we been honored and benefited by the presence of your Association. We will show the public the vast importance of our profession and its strength, and we know that when you close your meeting and leave us you will be glad that you came to the Garden State.

New Jersey veterinarians are becoming numerous, and the demand for the qualified veterinarian is greater than ever before. We have good representation in our ranks, and now that we have become well organized as a profession and have one strong State organization (the Veterinary Medical Association of New Jersey) we feel that we are on the threshold of advancement. We expect great good to come from our Association. We have several important committees—a Public Health Committee and an Animal Industry Committee. The scope of usefulness of these two committees is very broad. The Legislative Committee has much to do in the near future. We want to have a State Board of Veterinary Medical Examiners. Under authority of our Association there has been compiled and published the *Veterinary Medical Register of New Jersey*. Reference to this register will show that there are many men illegally registered in New Jersey, and at the last meeting of our State Association it was voted that the Committee on Legislation be authorized to take action against these men for illegal registering.

His Honor, the Governor of New Jersey, has recognized and complimented the veterinary profession by appointing two of its members on the "Good Roads Commission."

The control of the contagious diseases of animals is in the hands of the State Board of Health, with the exception of tuberculosis. A Special Commission on Tuberculosis has been established by authority of legislative enactment. This commission has done efficient work in exterminating tuberculosis in many dairies. The tuberculosis test is now required in all cattle shipped into the State, and if the testing is carefully done it will prevent the introduction of so much of this disease.

### Report of Resident State Secretary of Mississippi.

BY J. C. ROBERTS, AGRICULTURAL COLLEGE, MISSISSIPPI.

As Resident Secretary for Mississippi I have the pleasure of making the following report: As a whole, public sentiment regarding veterinary work has improved, and our State offers a more inviting field than ever for the practice of veterinary medicine. The main contagious diseases to which our attention has been directed have been anthrax, Texas fever, blackleg, and glanders. Of these, anthrax has been most destructive to our live-stock. An extremely fatal epidemic of this disease existed during the months of June, July, and August, causing a loss of probably not less than a quarter of a million dollars.

The absence of efficient State sanitary regulations prohibited our getting the disease checked at an early date; however, cremation, isolation, disinfection, and vaccination (where we were able to secure it) proved an efficient means of suppressing and preventing it. We have recommended that everyone having live-stock in infected districts vaccinate them early next spring—not later than the middle of next April.

### PROPOSED AMENDMENT.

To amend Article XI., of Chapter V., to read as follows: The Committee on Intelligence and Education shall collect and distribute among the members of this Association, on request, literature calculated to develop public interest in veterinary sanitary work and in veterinary legislation, municipal, State, and national, as to them seem best and in so far as the fund appropriated will admit, and also to report recent veterinary facts and intelligence.

